# Can Artificial Intelligence Revolutionize the Diagnosis and Management of the Atrial Septal Defect in Children?

**DOI:** 10.3390/diagnostics14020132

**Published:** 2024-01-06

**Authors:** Eliza Cinteza, Corina Maria Vasile, Stefan Busnatu, Ionel Armat, Arsenie Dan Spinu, Radu Vatasescu, Gabriela Duica, Alin Nicolescu

**Affiliations:** 1 Department of Pediatrics, Faculty of Medicine, “Carol Davila” University of Medicine and Pharmacy, 020021 Bucharest, Romania; eliza.cinteza@umfcd.ro (E.C.);; 2 Pediatric Cardiology Department, “Marie Skolodowska Curie” Emergency Children’s Hospital, 041451 Bucharest, Romania; armat_95ionel@yahoo.com (I.A.); nicolescu_a@yahoo.com (A.N.); 3 Department of Pediatric and Adult Congenital Cardiology, University Hospital of Bordeaux, F-33600 Bordeaux, France; corina.vasile93@gmail.com; 4 Cardio-Thoracic Department, Faculty of Medicine, “Carol Davila” University of Medicine and Pharmacy, 020021 Bucharest, Romania; 5 Cardiology Department, “Prof. Dr. Bagdasar Arseni” Clinical Hospital, 041915 Bucharest, Romania; 6 “Dr. Carol Davila” Central Emergency University Military Hospital, 010825 Bucharest, Romania; arsenie.spinu@umfcd.ro; 7 Department 3, Faculty of Medicine, “Carol Davila” University of Medicine and Pharmacy, 020021 Bucharest, Romania; 8 Emergency Clinical Hospital, 014461 Bucharest, Romania

**Keywords:** artificial intelligence, atrial septal defect, interventional cardiology

## Abstract

Atrial septal defects (ASDs) present a significant healthcare challenge, demanding accurate and timely diagnosis and precise management to ensure optimal patient outcomes. Artificial intelligence (AI) applications in healthcare are rapidly evolving, offering promise for enhanced medical decision-making and patient care. In the context of cardiology, the integration of AI promises to provide more efficient and accurate diagnosis and personalized treatment strategies for ASD patients. In interventional cardiology, sometimes the lack of precise measurement of the cardiac rims evaluated by transthoracic echocardiography combined with the floppy aspect of the rims can mislead and result in complications. AI software can be created to generate responses for difficult tasks, like which device is the most suitable for different shapes and dimensions to prevent embolization or erosion. This paper reviews the current state of AI in healthcare and its applications in cardiology, emphasizing the specific opportunities and challenges in applying AI to ASD diagnosis and management. By exploring the capabilities and limitations of AI in ASD diagnosis and management. This paper highlights the evolution of medical practice towards a more AI-augmented future, demonstrating the capacity of AI to unlock new possibilities for healthcare professionals and patients alike.

## 1. Introduction

Recent advancements in artificial intelligence (AI) have profoundly affected echocardiography, potentially elevating diagnostic processes in echo laboratories and improving patient care. In general, AI refers to the use of computer programs to perform complex tasks. Three factors have led to the rapid advance in AI in the last decade in multiple fields: (1) the development of hardware, including affordable graphical processing units that can perform massive amounts of calculations simultaneously; (2) the increasing availability of “big data” for training AI systems; and (3) the application of complex AI algorithms like neural networks. A key component of AI is machine learning (ML). When used correctly, a machine learning program improves with experience, so it becomes more capable of achieving its goal over time. Deep learning (DL) is a particular kind of machine learning that involves building neural networks with several layers. With each successive layer, the input data can be interpreted and predicted in a more abstract and high-level way. Currently, neural networks represent the state of the art in medical AI. Neural networks apply to various tasks (e.g., convolutional networks in computer vision and recurrent networks in language processing). To explain the relationship between raw and labeled data, a neural network builds a complex model using labeled data [1]. AI has been demonstrated to enhance echocardiographic images, automate measurement processes, and provide accurate cardiovascular diagnoses. Additionally, incorporating AI into echocardiography and training laboratory professionals in AI is vital to its successful implementation in clinical practice. Adopting AI in echo labs can effectively address issues like workload challenges and inconsistencies in diagnostic accuracy, ultimately leading to increased operational efficiency and improved patient outcomes [2].

In 2017, it was estimated that about 1.8 per 100 live births were diagnosed with congenital heart disease (CHD) globally [3]. Among these, atrial septal defects (ASDs) stand as the most common CHD in adults and the second most frequent CHD in children, constituting about 10% of all cases [4]. This defect consists of abnormal communication between the two atria. The condition favors the creation of a left–right shunt, resulting in volume overloading of the right cavities and increased pulmonary blood flow. Long-term pulmonary pressure increases with the onset of pulmonary hypertension, usually around the fourth or fifth decade of life, depending on the severity of the defect.

Often asymptomatic, ASDs are usually discovered during routine echocardiographic exams, with early the identification of significant defects being critical for timely interventions to prevent severe complications and mortality [5]. Electrocardiography (ECG), along with a chest X-ray, may raise the suspicion of ASD as the nonspecific primary tool for diagnosis. ECG may show a right-axis deviation, signs of right-atrial and -ventricle enlargement, incomplete right-bundle branch block, a tall P wave, rsR’ in V1, a tall R’, a deep S wave in V5-V6, notched R wave in leads II and III, and AVF. Atrial tachyarrhythmias may also be part of the clinical scenario in an ASD. The ECG changes can even be detected with good accuracy by AI programs. A chest X-ray may show cardiomegaly (dilatation of the right atrium and right ventricle), an enlarged middle left segment of the heart corresponding to the main pulmonary artery, with signs of increased pulmonary blood flow [6,7].

Transthoracic echocardiography (TTE) with Doppler flow imaging is the primary specific noninvasive technique for ASD detection, particularly in pediatric cases [8]. In addition to mere detection, TTE is capable of quantifying ASDs, assessing shunt direction and magnitude, evaluating changes in cardiac chamber sizes and functions, and abnormal pulmonary circulation pressures and flows [9]. However, TTE’s efficacy heavily depends on the expertise of well-trained physicians, who are often scarce, especially in remote regions [10]. The low disease prevalence, variability in image quality and interpretation, and limited views obtained during TTE contribute to its suboptimal sensitivity and specificity in ASD detection [11], thus impacting treatment referrals.

A hemodynamically significant defect can be closed surgically or through interventional procedures if spontaneous closure is assumed to be less possible [12,13]. Although surgical closure has almost zero mortality, a growing number of elite centers offer interventional closure with devices to over 80% of ASD patients in the interest of quick recovery and good results, as well as aesthetic and psychological benefits [14]. The pediatric interventional closure guidelines recommend interventional closure of the ASD in children weighing more than 15 kg [12]. Even children under two years old with a higher risk of major complications can undergo the procedure [15,16]. The incidence of major complications is almost five times higher in children under 15 kg (9%), including cardiac arrhythmias, transient atrioventricular blocks, and moderate mitral regurgitation [16,17].

**Figure 1:**
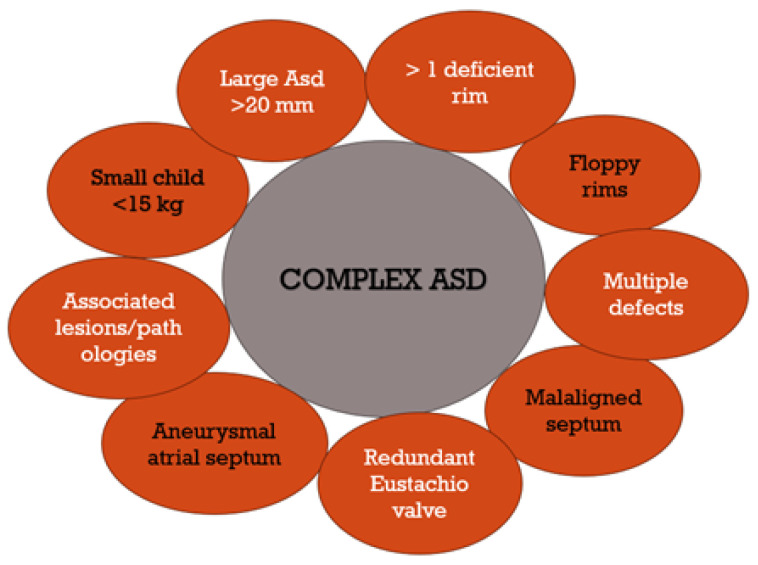
Complex ASD, for which interventional closure may be associated with failure and complications.

Anatomically, atrial septal defects are classified into four types with five locations: inferior and superior sinus venosus, coronary sinus, ostium secundum, and ostium primum; however, only two of them can be closed interventionally—the ostium secundum ASD type and the superior sinus venosus ASD type [18,19]. There are only a small percentage of superior sinus venosus ASDs (5–10%), and the practice of interventional closure is relatively new for this type of ASD [20]. Contrary to this, interventional closures of the septum secundum defect, which account for 75% of all cases, have already been performed for almost 50 years, with the first successful closure occurring in 1976 by King [21], and several months later, a second type of device was used by Rashkind [22]. In these devices with double discs, the delivery mechanism is the most significant difference [23]. Despite this vast experience, there are still many uncertainties and surprises in the successful implementation of an interventional ASD closure procedure due to the variable anatomy of the ostium secundum atrial septal defect, the anatomical apposition, the shape of the defect, the soft, floppy edges, and other peculiarities such as septal malalignment, the presence of a redundant Eustachian valve, or associated lesions (Figure 1) [14,24,25,26]. The measuring balloon can help anticipate the size of the device to be used. Still, some authors prefer adding 2–4 mm to the transthoracic echocardiography measurement, depending on the edges’ size and appearance/consistency.

Transesophageal echocardiography (TEE) is an excellent guide for transcatheter implantation in ostium secundum ASD, especially when implanting multiple devices simultaneously in small children or complex cases. With the help of three-dimensional (3D) Doppler TEE, one can determine the spatial relationship between multiple defects (round, racquet, oval, or star) and make device selection decisions [27,28,29]. In many cases, generating high-resolution 3D images with optimal two-dimensional (2D) images may only be possible.

With a 100% success rate and a 2% major complication rate, intracardiac echocardiography may be the best choice during transcatheter device implantation. Due to its cost and the need for a second vascular access of at least 8F, this procedure is not recommended even in elderly children [30,31].

Per the 2015 guidelines of the American Society of Echocardiography (ASE), echocardiography plays a crucial role in determining the treatment approach for atrial septal defects (ASDs), including the decision between transcatheter and surgical interventions. In the study of echocardiographic, color flow Doppler echocardiography is the most precise way to estimate the ASD diameter, as measured during surgery. This method is more accurate compared to relying solely on standard 2D echocardiograms, which can lead to significant measurement errors [32].

Deep learning models have been increasingly utilized to address these challenges for the automated detection and assessment of cardiovascular diseases using echocardiographic images and videos. These models can perform multiple tasks, including image quality assessment, view classification, boundary segmentation, disease diagnosis, and automatic quantification [33,34,35,36,37]. AI’s ability to predict the dimensions of closure devices could significantly improve ASD management, resulting in fewer shunts, embolisms, erosions, and cardiac tamponades. By leveraging AI, a new frontier in the interventional closure of ASD could be opened, marked by increased precision, reduced risks, and improved patient outcomes.

## 2. The Evolution of AI in Echocardiography

Over the past decade, the use of AI in cardiovascular research has grown remarkably. Artificial intelligence algorithms have been widely implemented in areas such as image-based diagnosis, image segmentation and reconstruction, and image quality enhancement. In addition, cardiology has embraced AI-based devices and software tools for evaluating risk [38].

TTE, characterized by its accessibility and widespread use, is a fundamental imaging technique offering real-time cardiac visualization and immediate identification of structural anomalies. AI enhances the precision of imaging measurements by reducing variability between and within operators and revealing details too subtle for human detection (Table 1). Machine learning (ML) algorithms have been extensively utilized in TTE for tasks such as image-based diagnostics, segmentation, and patient prognostication [39].

A notable innovation in 2D TTE image analysis employs AI for pattern recognition, automating the calculation of the left-ventricular ejection fraction (LVEF). This AI system produces results comparable to standard manual estimation methods (biplane Simpson’s method) with reduced variability compared to traditional visual EF assessments [40]. Another multicenter study explored a fully automated computer vision software, AutoLV, TomTec-Arena 1.2, TomTec Imaging Systems (Unterschleissheim, Germany), employing ML for measuring left-ventricular volumes, EF, and average biplane longitudinal strain (LS). This automated approach, achieving measurements in 98% of cases with an average processing time of 8 s per patient, demonstrated an efficient method for LVEF and LS assessment [41].

A groundbreaking study involved automated echocardiogram interpretation in clinical settings using ML. A convolutional neural network (CNN) trained on 14,035 echocardiograms with 70,000 pre-processed images was used to identify 23 viewpoints and segment cardiac chambers across 5 common views. The CNN processed grayscale images through multiple convolution and pooling layers, culminating in a 23-way softmax output layer representing different echocardiography views. This study established that automated measurements were on par with or superior to manual assessments across various internal consistency metrics [33]. In another significant study, a CNN capable of classifying 15 standard echocardiography views with high accuracy was developed, surpassing the accuracy of board-certified echocardiographers in some instances [42].

In cardiomyopathy, ML algorithms trained on clinical and conventional echocardiography data, including speckle-tracking variables, effectively differentiated constrictive pericarditis from restrictive cardiomyopathy. The associative memory classifier (AMC) demonstrated excellent performance with an area under the curve (AUC) of 89.2%, outperforming common echocardiography variables for distinguishing these two similar conditions [43]. An ensemble ML model combining support vector machine (SVM), random forest (RF), and multilayer perceptron (MLP) algorithms utilized speckle-tracking echocardiographic data to differentiate inherited hypertrophic cardiomyopathy (HCM) from physiological hypertrophy in athletes, showing superior sensitivity and specificity compared to standard diagnostic criteria [44].

AI methods have also been applied to valvular diseases, such as using SVM classifiers for the classification and severity assessment of mitral regurgitation (MR), achieving high sensitivity and specificity in distinguishing the severity of MR in normal subjects [45].

The most recent advancement in AI-enhanced echocardiography is EchoNet-Dynamic, a video-based deep learning (DL) algorithm that surpasses human expertise in tasks like EF estimation, cardiomyopathy assessment, and left-ventricle segmentation. This algorithm utilizes standard apical four-chamber echocardiogram videos with variance in predictions comparable to or less than human experts. It employs spatiotemporal convolutions with residual connections for EF prediction of each cardiac cycle, with frame-level semantic segmentations of the left ventricle generated through weak supervision from expert human tracing. EchoNet-Dynamic, trained on 10,030 apical four-chamber echocardiogram videos, represents the first video-based DL echocardiography model, showing enhanced EF measurement performance. Its rapid detection of subtle EF changes supports precise, real-time cardiovascular disease diagnosis [36].

A study by Vasile et al. tested the application of AI software (Heart Company, Vilnius, Lithuania), initially developed for adults, on pediatric echocardiograms. The study involved 45 children split into two groups: under 9 years and over 9 years. The results showed strong correlations between junior and senior cardiologists’ assessments across most parameters. However, the correlations between AI software and senior cardiologists were variable, with some discrepancies noted, especially in younger patients. The software performed better in the older age group, particularly in assessing the sinotubular junction and EF. Despite showing potential, the study highlighted the need for further software refinement for more accurate and consistent use in pediatric echocardiography [46].

In the context of periprocedural evaluations, AI has been employed to assess the aortic annulus in patients scheduled for transcatheter aortic valve replacement. A single-center investigation involving 47 patients compared aortic annular measurements obtained through AI software with those derived from traditional 2D transesophageal echocardiography and cardiac computed tomography. The AI software’s measurements demonstrated a strong correlation with the cardiac computed tomography data and surpassed the accuracy of TEE (correlation coefficient r = 0.84; *p* < 0.0001) [47].

## 3. Potential for AI in ASD Diagnosis

**Figure 2:**
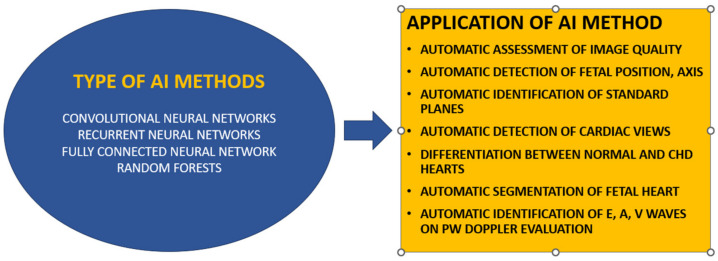
Type of AI methods used to obtain information in fetal echocardiography using DL models.

CHD can be better diagnosed prenatally using machine learning (Figure 2). To identify abnormalities within the cardiac anatomy of the fetal heart, Yeo et al. used an intelligent navigation method called FINE [48,49]. Several improvements and advancements have been made to this technology, including automatic fetal positioning, automatic cardiac axis, and cardiac biometry [50]. In a study involving 1003 healthy fetuses, Baumgartner et al. used CNN to automatically detect standard planes [51]. Le et al. also succeeded in differentiating between normal and CHD-affected fetuses on 3910 subjects, with a percentage of 14.1% CHD [52]. Also, pulsed Doppler screening on five healthy fetuses was performed automatically with AI model in a study conducted by Sulas [53]. An automated prenatal screening system based on DL also was introduced by Arnaout et al., in which 16 major CHDs were included. The CHD images were distinguished from normal images using CNN models trained to identify five standard views. A total of 4108 fetal surveys, including 400 fetal echocardiograms with CHD, were used to test the models developed. The DL model using fetal screening ultrasounds, focusing on tasks like view selection, segmentation, and the detection of complex CHD, was tested on 4108 fetal sonograms and achieved an area under the curve (AUC) of 0.99, effectively differentiating normal from abnormal hearts, a performance level on par with expert clinicians. The sensitivity of the model was 95%, while its specificity was 96%. The authors suggested that their algorithm would make prenatal CHD screening twice as effective [54].

AI solutions for pediatric echocardiography include four key steps: acquiring images, segmenting views, quantifying chambers, and interpreting images. A poorly compliant infant or child, or a less experienced operator, results in poor-quality images [55,56]. in one study, researchers trained a CNN to discriminate accurately between patients with discordant atrioventricular and ventriculoarterial connections post atrial switch procedures and normal controls using supervised deep learning [57]. Recent research has demonstrated the impressive capabilities of DL models in the diagnosis of ASDs. Zhao et al. created a modified version of the U-Net architecture for segmenting the atrial septum structure in magnetic resonance imaging of patients with pre- and post-occlusion ASDs, achieving a mean Dice index of 0.81 [58].

The accuracy and reliability of echocardiography assessments are essential for clinical decision-making. While echocardiographic images are difficult to acquire and analyze, image interpretation relies on experienced echocardiographers and fetal cardiologists. It may be possible to automate CHD assessment thanks to artificial intelligence, in particular deep learning. For DL models to perform accurate measurements and diagnostics, a training data set should be curated to build high-fidelity DL models based on images of high quality [55]. Wang et al. developed an end-to-end framework capable of automatically analyzing multi-view echocardiograms and selecting critical frames for disease diagnosis, successfully distinguishing between ASD, ventricular septal defect (VSD), and normal cases with 92.1% accuracy [59].

In a study performed by Hong on ASD detection using AI, an independent test set of 229 cases contained 203,619 images, of which 105 had ASD and 124 did not. The results were based on a training set of 4031 cases containing 370,057 echocardiographic images. In this study, a three-stage fully automatic system was used to detect ASDs. Using four echocardiographic views (subcostal view focusing on the atrium septum, apical four-chamber view, low-parasternal four-chamber view, and short-axis view), the ASD was first identified. In the second and third stages, the aim was to segment the target cardiac structure and detect and conclude the presence of ASD in the patient. Based on the proposed system, CHDs can be automatically and accurately diagnosed using artificial intelligence [60].

An impressive study, so far, is the recent one conducted by Lin et al. A deep learning framework was developed specifically for use with color Doppler echocardiography. This framework was designed to automatically detect and quantify atrial septal defects (ASDs), a notable advancement, since previous research had not automated the interpretation of color Doppler videos for this purpose [61].

The research performed by Lin et al. involved a substantial dataset comprising 821 echocardiographic examinations from two tertiary care hospitals. These examinations were used as both the training and external testing datasets. The DL models created were capable of processing color Doppler echocardiograms automatically. This included critical tasks such as view selection, ASD detection, and pinpointing the atrial septum’s endpoints and the defect itself. These capabilities were essential for quantifying the size of the defect and the residual rim. The view selection model demonstrated an exceptional average accuracy rate of 99%, efficiently identifying the four standard views necessary for ASD evaluation. For ASD detection in the external testing dataset, the model achieved an area under the curve (AUC) of 0.92. This was accompanied by a sensitivity of 88% and a specificity of 89%. The model’s ability to automatically measure the defect size and residual rim was accurate, with mean biases of only 1.9 mm and 2.2 mm, respectively. The study concludes by underscoring the feasibility and effectiveness of using a DL model for the automated detection and quantification of ASDs via color Doppler echocardiography. This technological advancement promises to improve the accuracy and efficiency of color Doppler in clinical practice, especially for screening and quantifying ASDs, which are critical for informed clinical decision-making [61].

## 4. Challenges and Limitations

Identifying the 20% of cases that are not suitable for interventional closure of the ostium secundum ASD is a challenging task. There are many limitations in the processes of decision-making for interventional closure, and some of the main issues are presented in Table 2.

Rim deficiencies associated or not with aneurysmal tissue, large defects, multiple defects, and enlargement of the defect by the balloon sizing technique in closing the ASD are issues of debate in congenital interventional cardiology, both in children and adults, and they represent challenges or limitations for closure procedures. We are balancing the risk of device migration, which increases for rim deficiency, and the risk of cardiac erosions by oversizing the device’s dimension. There may also be a floppy posteroinferior rim in about 25% of ASDs, defined as moving backward and forwards with blood flow. To avoid migration, a slight oversizing is permissible in general. To select the correct device, measurements performed by TTE, TEE, or balloon sizing must be adjusted. AI programs may help determine appropriate patients for interventional closure and predict the correct device dimension for such complex cases.

Researchers have found that patients presenting a posteroinferior rim deficiency are associated with both closure failure and concurrent adverse events [62]. Among the 474 patients in Kijima et al.’s study, 35 had deficits of more than one rim (7.4%). Most of these patients had a significant deficit in the aortic and posteroinferior rim. This group had a lower success rate for percutaneous closure (86%) than patients with sufficient rims (100%) or patients with only one rim deficit (98%) [63]. Regarding the deficient posteroinferior rim, in their study, Cao et al. were able to treat patients with less than 5 mm rims using devices up to 6 mm larger than the maximum measured diameter, and they monitored the rims using TTE and TEE simultaneously [64]. On the contrary, in a study by Huang, the same success rate was achieved in both groups with sufficient and deficient posteroinferior or inferior vena cava rim [65]. Occlusion by percutaneous means may be difficult in large defects with floppy posterior or posteroinferior edges [62,66,67].

A large ASD can be defined as a ratio >15 mm/m^2^ between the ASD diameter and the body surface area (BSA) [68] or as 1.2 times the ASD diameter by weight [16]. A poor anterior, posterior, or superior rim is also a risk factor for unfavorable outcomes [16,68,69,70]. Lahiri found that ASDs larger than 20 mm with deficient posterior, posterosuperior, and inferior rims experienced unsuccessful closure [70]. With the oversizing of the device, complications such as aortic erosion, cardiac perforation, and atrioventricular block may arise [16,26,68,69,71]. In 1% of cases, device embolization occurs due to undersizing, soft, floppy rims, or reduced operating experience and is usually caused by undersizing of the defect. A displacement can occur immediately or within 24 h but has also been described as occurring late [72]. For one or two deficient rims, authors recommend oversizing by 2 or 4 mm, normally less than 20–30% of the measured diameter, either by TEE or balloon sizing [69,73].

About 10 to 15% of ASDs have multiple fenestrations [26]. In most cases, they are associated with an aneurysmal atrial septum, characterized by an excursion greater than 10 mm. Children with large ASDs and septal aneurysms should be analyzed using the total septum length (TLS) measured in a four-chamber view rather than balloon sizing [71]. An appropriate TLS should exceed the left-side device diameter [71]. There is substantial debate in the medical literature about whether a single device could be used or if multiple devices are required. Each of the options has pros and cons. Using only one device may achieve a shorter procedure time, with less time spent exposed to X-rays, lower erosion and embolization risks, and greater financial advantages [74]. It is more difficult to perform the procedure when using multiple devices, and there is a higher risk of embolization or erosion when using multiple devices [75]. Using more than one device on 33 patients, Awad et al. reported a 6% complication rate (one embolization case and one erosion case) and a 97% immediate success. The study considered a distance greater than 7 mm to be optimal for implanting two devices. Multiple device implantation requires closing the larger defect first [75], but the procedure may differ from school to school. In one report, 19 of 34 children with multiple ASDs were treated with just one device (54.9%), and 15 children received more than one device (two devices for 14 patients and three devices for 1 patient). According to the report, the patient who received three devices (31 years old) died at home 30 days after the procedure with cardiac tamponade without clinical signs of erosion [76]. Usually, cardiovascular erosion occurs within five days from the implantation procedure in 75% of cases. In another study involving 148 patients with multiple ASDs, Masseli et al. reported almost the same percentage (63.5%) for a single device in the case of multiple defects [77]. Among 83 patients with complex ASD anatomy, Santoro et al. reported a 95.8% use of a single device for complex ASD closure [78]. According to some authors, the Amplazer Cribriform Occluder is not ideal for the closure of aneurysmal multi-fenestrated ASDs, since its waist (connecting pin) is narrow and cannot accommodate a large device [79].

Balloon sizing of the septal defect has long been considered the gold standard for determining device size. However, it is associated with its own set of limitations and complications. Balloon sizing can cause overstretching of the defect rims, which increases the defect size, leading to the use of an oversized device [80]. Stretching of the defect rims can lead to an inadvertent tear of the atrial septum, micro-embolization causing stroke, and balloon-related cardiac perforation [81]. Therefore, many operators have foregone balloon sizing of ASDs for imaging-guided ASD closure. The balloon sizing technique is also used for multifenestrated ASDs. Once the left-to-right flow disappears, a second balloon is inflated, and if the distance between the two openings is less than 5–7 mm, one occluder is used [26,75,82]. To avoid device erosion, 2 mm should be added to the diameter obtained by balloon sizing at stop-flow. The measurement should be smaller than the total septum measured in the four-chamber view [71]. In their studies, Carano et al. suggested that atrial balloon septostomies may be performed in cases where large or two to three smaller devices cannot close the septum [83]. However, the risk of obtaining an unpredictably enlarged hole, which can interfere with the subsequent interventional closure of the new defect, remains. A study of 161 children was conducted by Baruteau et al. on the effect of balloon stretching on the largest ASD among multifenestrated aneurysmal ASDs, and the conclusion was that the balloon-stretched diameter increased from 15 mm/m^2^ (TEE)–17 mm/m^2^ (TTE) to 26.3 ± 6.3 mm/m^2^ after inflation. With a defect stretching to 26.3 mm × 6.3 mm/m^2^, Baruteau et al. achieved an estimated closure rate of 92.6% [68]. Another study compared balloon sizing with TEE. A total of 79.7% of patients in group I had success with balloon sizing via fluoroscopy, compared with 89.6% who had TEE guidance for the closure of the ASD in group II. ASD device upsizing was significantly lower in group II (*p* = 0.001). Also, TEE showed higher success rates with smaller devices in patients with large ASDs (>25 mm) and those under 14. A total of four cases of device embolization were reported (two in each group); one death occurred in group II despite successful surgical retrieval [84].

The routine use of transesophageal echocardiography (TEE) has led to a better understanding of the anatomy of ASDs and plays a vital role in determining device size [85]. Using 2D TEE, defects can be assessed, and the appropriate device can be selected. Nevertheless, 2D TEE measurements may be inconsistent, especially in complex ASD, depending on the plane of the image obtained. Due to its inherent limitations, a 3D structure cannot be visualized in a 2D plane. Three-dimensional imaging provides a better understanding of cardiac structures than 2D echocardiography [86]. Three-dimensional TEE allows for the assessment of the rims of ASDs as well as an understanding of their spatial orientation [87]. In recent studies, 3D TEE is as safe and effective as balloon sizing in the assessment of ASDs [88]. ASD characteristics can be similarly assessed with both 3D TEE and 2D TEE. An evaluation of 30 ASD patients using 2D and 3D TEE was conducted by Hascoet et al., and the dimensions of the ASDs were compared with balloon diameters based on the measurements taken. Both techniques showed a high equal correlation for round and oval ASDs [89].

Despite the beneficial effects of 2D TTE, 3D TEE, and balloon sizing, interventional closure in ASDs can be accompanied by complications, particularly in complex cases where 2D TTE, 3D TEE, and balloon sizing are insufficient to ensure a successful outcome. Depending on the location, size, number of fenestrations, hemodynamic consequences, associated lesions, treatment difficulties, and the patient’s age, any of them can be considered a complex ASD [8,14,90,91,92]. In some cases, ASD closure may be performed using a fenestrated device in patients with pulmonary hypertension and diastolic dysfunction [74,83,91].

It is possible to face rhythm and conduction disturbances after implanting a device (1–4%). A device causing atrioventricular block (AVB) must be removed if the AVB persists after corticoid therapy. Posteroinferior margin deficiency, the misuse of large devices, and preexisting conduction abnormalities are risk factors for complete AVBs [14]. In addition to tissue dissection, oversized devices on a floppy atrial septum may cause arrhythmias [74].

Cardiac erosions are a serious issue and appear in 0.1–0.3% of cases, usually early after the procedure (within one year). However, they may also occur late [93,94,95,96,97]. There is a high incidence of damage to the walls of the right or left atriums or the junction between the right and left atriums and the aorta. Aortic fistulas, hemopericardium, and pericardial tamponade can be caused by erosion and perforation [98]. Approximately 10% of patients who develop this complication die if emergency cardiac surgery is not performed urgently [95]. A large device, inadequate anterior and superior rims, repeated attempts to position the device, the type of occluder (Amplatzer, Occlutech, and Cardia), adult age, and the device’s movement into the heart may be associated with cardiac erosion. Identifying defect areas by 3D TEE can also help reduce oversizing and complications related to large devices, such as mushrooming and cardiac erosion [95].

Integrating AI in healthcare, particularly in diagnosing and managing ASDs, brings a set of ethical, legal, and privacy concerns. Ethically, AI must ensure patient autonomy, consent, and confidentiality, especially when dealing with sensitive health data. Legally, there are concerns about liability and accountability, particularly diagnostic errors or mismanagement due to AI decision-making. Privacy issues stem from handling and protecting patient data within AI systems, highlighting the need for robust data security measures.

Furthermore, the current AI technologies in ASD diagnosis and management face limitations, especially in complex cases. The accuracy of AI models is highly dependent on the quality and diversity of the training data, which can impact their effectiveness in varied clinical settings. Integrating AI tools into existing healthcare systems is also challenging, which may require significant infrastructure changes and substantial investment. Another limitation is the potential for AI algorithms to perpetuate existing biases, leading to unequal care or misdiagnosis in underrepresented populations.

**Table 2:** Challenges and limitations in ASD diagnosis and management.

Study	Challenge or Limitation	Description
Contreras [62], Kijima [63]	Posteroinferior rim deficiency	Associated with both closure failure and concurrent adverse events. Patients with this deficiency have a lower success rate compared to those with sufficient rims.
Cao [64], Huang [65]	Device selection and measurement	Correct device size selection is crucial. In cases of rim deficiency, measurements need adjustment, and slight oversizing is permissible to avoid migration.
Carlson [80], Harikrishnan [81]	Complications of balloon sizing	Balloon sizing can cause overstretching of defect rims, leading to the use of oversized devices and potential complications such as atrial septum tear, stroke, and cardiac perforation.
Rana [85], Mor-Avi [86], Taniguchi [87]	Use of transesophageal echocardiography (TEE)	TEE, including 3D TEE, provides a better understanding of ASD anatomy and plays a vital role in determining device size. It has been found to be as effective as balloon sizing.
Mahmoud [14], Shuler [90], Bartakian [8]	Complex ASD cases	Complications in complex ASD cases where traditional methods are insufficient. Includes factors like location, size, number of fenestrations, associated lesions, and patient age.
Butera [26], Amin [71], Awad [75]	Multi-fenestrated ASDs	Challenges in treating ASDs with multiple fenestrations, requiring consideration of aneurysmal septums, floppy rims, heart dilatation, and potentially multiple devices.
Faccini [93], Kim [94]	Ethical, legal, and privacy concerns in AI use	Ensuring patient autonomy, consent, and confidentiality in AI use. Legal concerns about liability in diagnostic errors or mismanagement. Privacy issues in handling patient data.
Tal [74], Amin [95]	Limitations of AI technology	AI model accuracy depends on training data quality and diversity. Challenges in integrating AI into existing healthcare systems and potential for bias in underrepresented populations.

## 5. Future Research Directions

Complex cases may benefit from specific calculations and measurements. Different ratios and equations were calculated to predict a good result for device implantation. It may be useful to use a ratio of 0.35 for the defect/total septum, 0.75 for the superoanterior rim to the defect size, or 1 for the inferoposterior rim to the defect size as a predictive tool [99].

Several studies have found formulas to predict the device size to be used based on different echocardiographic measurements (Table 3). Planning for device closure and sizing can be made easier by incorporating the 2D/3D characteristics of the defect after these correlations. Two-dimensional-TEE-based ASD sizing is considered the gold standard in many institutions for percutaneous closure, especially if routine balloon sizing is not performed. A lot of information might be provided by 3D TEE features such as the defect area and defect circumference. A regression equation can be applied to the 3D defect area and circumference measured by TEE, with no differences between round and oval shapes [79]. According to Seo et al., measured diameters of balloon-stretched ASDs and diameters measured using 2D and 3D TEE showed a close correlation [100]. In the study performed by Mani et al., it was found that the device size can be determined more accurately by evaluating the defect area and circumference on 3D TEE than by measuring diameters [92].

According to Seo et al., the equation derived by them was validated in a large study of 250 patients, in which the diameter of the initially estimated device was compared with the diameter of the balloon-stretched ASD. Using 3D-TEE-derived data from the second tier of the study, the device size for percutaneous closure was calculated without considering balloon sizing. A 99% procedural success rate has been observed in patients who have undergone device closure only based on 3D TEE data. During percutaneous ASD closure, 3D TEE alone shows safety and accuracy. It is recommended to avoid balloon sizing of ASDs since it can lead to an overestimate of the device size [92,100].

Hascoet and colleagues also devised models for predicting the balloon-stretched diameter based on 3D ASD measurements. One model included the 3D diameter, while the other included the area. In this study, two separate models were used to predict the stretched diameter of the balloon rather than the actual device size [89]. Roushdy et al. devised a novel method of transcatheter device closure using a 3D echocardiographic method. The ASD’s 3D area and circumference were used to predict device size in two models [101]. Complex models are difficult to use in clinical practice. Furthermore, routine balloon sizing is unnecessary for ASD device closure, and TEE alone can produce better results.

Looking backward, AI may provide algorithms for pointed measurements and calculations and may also provide an estimated device size, taking into consideration the area, the circumference, the diameters, and other bad prognosis factors, such as the dimensions of the specific rims, floppy rims, and aneurysmal septums. AI in diagnosing and managing ASD is poised for significant growth and innovation. Future developments may focus on creating more sophisticated algorithms that handle complex diagnostic scenarios, including integrating AI with other emerging technologies. This could lead to more accurate, individualized treatment plans and improved patient outcomes.

Research and development in this field could also explore creating more inclusive AI models trained on diverse datasets, reducing bias, and improving diagnostic accuracy. There is potential in enhancing the interoperability of AI systems, allowing for seamless integration with various electronic health records systems and diagnostic tools.

Moreover, future research might address the ethical, legal, and privacy concerns associated with AI in healthcare. This includes developing standardized guidelines for the ethical use of AI, establishing clear legal frameworks for liability and consent, and implementing robust data security measures to protect patient privacy.

In summary, while AI in ASD diagnosis and management faces challenges, the prospects are promising, with ample opportunities for research and development to enhance its efficacy and integration into healthcare systems.

Certainly, exploring the intervariability between the predicted size of the device based on measurements from TTE, transesophageal echocardiography, invasive balloon sizing, and AI algorithms, compared to the actual size of the device used for closure, could be an interesting avenue of research.

## 6. Conclusions

This review underscores the significant potential of AI in revolutionizing the diagnosis and management of ASDs. AI’s role in enhancing echocardiographic accuracy and automating diagnostic processes promises to improve patient outcomes in cardiac care. However, this advancement comes with challenges, including ethical considerations, data privacy concerns, and the need for AI to integrate seamlessly into existing healthcare systems.

## Figures and Tables

**Table 1:** Comparison of traditional vs. AI-enhanced diagnostic methods.

Method	Traditional TTE	AI-Enhanced Echocardiography
Description	Primary noninvasive technique for ASD detection.	Reduces variability between operators, reveals subtle details, and automates diagnostics and patient prognostication.
Dependency on expertise	Efficacy depends on the expertise of physicians.	Less dependent on operator expertise.
Accuracy and efficiency	Varies based on physician skill and experience.	Consistently high accuracy and efficiency.

**Table 3:** Prediction formulas for selecting the correct device size.

Nr	Formulas for Predicted Device Size	Author
1	(0.964 × 3D Maximum Diameter) − (2.622 × Circular Index) + 7.084	Seo et al. [100]
2	0.03199 (3D Defect Area) + 0.01238 (3D Defect Circumference) + 17.39961	Mani et al. [92]
3	10.8 + (3.95 × 3D ASD Area) (3.85 × 3D ASD Circumference) − 1.02	Roushdy et al. [101]
4	1.07 × 3D-TEE(max) − 3.1 × ASDshape + 3; ASDshape circular 0; ASDoval 1 4.5 × ASDarea + 11.5	Hascoet et al. [89]

## Data Availability

Data are contained within the manuscript.
